# Practical Notes on Diagnosis and Treatment

**Published:** 1912-02-17

**Authors:** 


### PRACTICAL NOTES ON DIAGNOSIS AND TREATMENT.

**Novoform.**

This substance has the antiseptic and other surgical attractions of iodoform, but is entirely free from odour. Hence as a dressing for wounds it has manifest advantages.

**A Pill for Chronic Constipation.**

The following is a useful formula: Pil. Aloes et Ferri, Pil. Rhei Co., Ext. Cascar. Sagrad., a 1 grain, Ext. Belladon. Alcohol gr. $\frac{1}{8}$. One or two every night at bed-time.

**Foreign Bodies in the Cornea.**

After removing a particle of dust or other foreign body from the cornea a drop of atropine solution should be instilled, and the eye should be covered with a pad and bandage for twenty-four hours. Rest is needed in order that the abraded corneal surface may be healed over. If the procedure here advised is not followed, the patient continues to suffer discomfort and is apt to believe that the foreign body has not been removed.

**Belladonna in Local Inflammations.**

Belladonna, if thoroughly applied in the earlier stage of local inflammations, *e.g.* adenitis, tonsillitis, appendicitis, etc., will often check the inflammatory process and prevent suppuration. A suitable preparation is alcoholic extract of belladonna, 2 parts; lanoline and vaseline, of each 1 part. This should be gently but perseveringly rubbed into the skin, and a poultice should then be applied, the belladonna application being repeated before each fresh poultice.

**Changes in the Fundus Oculi.**

The existence of good vision is quite consistent with serious organic changes in the fundus oculi. Optic neuritis, in cases of cerebral tumour, for example, may be present although the patient makes no complaint of defective sight. And the same is true of retinal hæmorrhages and retinitis occurring in chronic nephritis or glycosuria. Even primary optic atrophy, as in tabes dorsalis, may in its early stages fail to interfere with central vision, though, if the field of vision is taken, this may be found to show some degree of contraction.

**To Measure the Urine.**

In obtaining a measure of the twenty-four hours volume of urine it is necessary to give the patient exact instructions, or the observation will probably be inaccurate. Without direction he will almost certainly collect all the urine from, say, 8 A.M. on one day to the same hour on the next. But this, manifestly, will be more than the amount excreted by the kidneys in twenty-four hours, as it will include the quantity collected in the bladder prior to the commencement of the selected period. The better way is to tell the patient to pass urine at, say, 8 A.M., and to throw this away, and then to collect all the urine that is passed up to and inclusive of 8 A.M. on the following day.

**Urticaria.**

A solution of benzoic acid in alcohol (1 in 20) used as an evaporating lotion is a grateful application to relieve the irritation of urticaria.

**Retinal Detachment.**

Sir Wm. Whitla has found in some cases that this very serious condition has shown marked improvement under the use of full hypodermic doses of pilocarpine.

**Post-Herpetic Pain.**

It is a commonplace experience that the pain following herpes in adults is often very difficult to cure. The remedies recommended are legion. One which is sometimes forgotten is colchicum; it occasionally acts very beneficially. Another helpful remedy is cod-liver oil. And in some instances the continuous current is successful. Another local agent is the compound salicylate of methyl ointment of the British Pharmaceutical Codex.

**A Soothing Ointment.**

A very soothing application, suitable for an acute erythema or eczema, consists of vaseline, zinc ointment, and liniment of lime. It should be applied, spread thickly, on strips of lint. An important factor in the treatment of any acute dermatitis is to abstain from washing with soap and water. The ointment, before another application is made, should simply be wiped off with a soft linen cloth, or the skin may be gently cleansed by using a fine sponge dipped in milk.

**Poisoning by Ammonia.**

This sometimes occurs as the result of the accidental administration of one or other of the domestic preparations of ammonia. The immediate claim is, of course, for a dilute acid. Vinegar, lemon-juice, citric acid, or tartaric acid are generally at hand. It is especially necessary to remember that as a consequence of inhalation of the fumes there may be, even after some hours, serious laryngeal symptoms and œdema glottidis, and these may call for steam inhalations, cocaine spray, and even tracheotomy.

